# Association between *TNF-α*, *Interleukin-18* Polymorphisms and Risk of Hepatocellular Carcinoma in Egyptian patients

**DOI:** 10.31557/APJCP.2021.22.3.887

**Published:** 2021-03

**Authors:** Heba Sharafelldin, Abdalla Morsy, Hany Elghobary, Enas Osman, Normeen Rady

**Affiliations:** 1 *Clinical and Chemical Pathology, Cairo University, Cairo, Egypt. *; 2 *Clinical and Chemical Pathology, Theodor Bilharz Research Institute, Cairo, Egypt. *

**Keywords:** TNF-α, HCC, interleukin- 18, polymorphisms-SNP, real time PCR

## Abstract

**Objective:**

To evaluate the association of gene polymorphisms of the *SNP* of *TNF-α* gene *-238G>A* and* IL-18 *gene-*607C>A* with the development of hepatocellular carcinoma among Egyptian patients.

**Methods:**

One hundred and fifty patients were allocated to this study; eighty patients with hepatocellular carcinoma (Group A), seventy cancer-free HCV age, and sex-matched patients (Group B). We analyzed two Single nucleotide polymorphisms (SNPs)* (TNF-α-238G>A* and* IL-18-607C>A)* by real-time polymerase chain reaction using sequence-specific primers (PCR-SSP).

**Results:**

Significant higher risk of HCC was associated with genotype* IL-18–607AA* (P<0.001), OR: 5(2.188-11.47), allele *IL-18 -607⁄A* (P=0.001), OR: 2.1(1.32-3.3). A significant association was found between the size of HFL in the HCC group and different genotypes of *IL18 *genes (P=0.013) where 62.5% of patients with tumor size >5 cm carried the risky (AA) genotype on the other hand the* SNP* of *TNF-α* gene *-238G>A* showed no statistically significant association between the two groups.

**Conclusion:**

The SNP -607C>A in the IL18 gene was associated with increased HCC risk in Egyptian patients suggesting its use as a potential diagnostic non-invasive tool that allows to identify a new group of HCC patients at an earlier stage.

## Introduction

The majority of HCC cases occur in patients with cirrhosis or with chronic liver diseases resulting in the release of inflammatory mediators causing cell injury followed by regeneration mediated by the immune response which plays a major role in hepatocarcinogenesis (Choi and Lim., 2017). Among inflammatory mediators, tumor necrosis factor-α (TNF-α) which been implicated in inflammation-associated tumors (Teixeira et al., 2013). The TNF-α protein induces the expression of adhesion molecules, facilitating the invasion of metastatic tumor cells, the observation of high levels of TNF-α have been recorded in the blood of some cancer patients (Wang et al., 2010). The overall expression and secretion of cytokines can be affected by many genetic polymorphisms in the promoter region of TNF-α, among them TNFα-238G>A polymorphism (Shin et al., 2015). TNFα-238G>A polymorphism has been reported to alter the risk of several types of cancers, such as HCC, breast cancer, lung cancer, non-Hodgkin lymphomas, and prostate cancer (Ma et al., 2014).Interleukin-18 (IL-18) belongs to the IL-1 cytokine superfamily and it is a novel proinflammatory cytokine. It was initially recognized as an interferon-γ (IFN-γ)-inducing factor and has a wide range of functions, including induction of the synthesis of IFN-γ in T cells and natural killer cells through synergistic action with IL-12 and IL-10, respectively, promotion of Th1-type immune responses, and enhancement of the proliferative response and cytokine production of activated T cells (Zhang et al., 2016). As HCC is a typical inflammation-related cancer, these biologic effects of IL-18 might thus be implicated with HCC development, suggesting that abnormal expression of IL-18 could be associated with the pathogenesis of this disease (Yang et al., 2013). Several SNPs in the promoter region and two polymorphisms in the 5′-nontranslated region of IL-18 have been identified, among these, the functional polymorphism in the promoter region at loci −607C>A the most studied (Jia et al., 2016).

## Materials and Methods

This case-control study was conducted on a total number of one hundred and fifty subjects; they were recruited from the tropical medicine department, Theodor Bilharz Research Institute. All patients were subjected to full history taking, full clinical examination. They were divided into two groups, Group (A) Included eighty patients with hepatocellular carcinoma, 49 males and 31 females, with a mean age of 62.15±7.5 years. Group (B) included seventy patients with cancer-free HCV age and sex-matched patients, 42 males and 28 females with a mean age of 56.47±9.35 years. HCC patients diagnosed by clinical examination, serum AFP level and typical findings on abdominal ultrasound, and abdominal triphasic CT scan; HCV patients diagnosed by clinical examination, anti-HCV Abs, and ultrasonographic criteria. All patients were free from any inflammatory conditions as spontaneous bacterial peritonitis, inflammatory bowel diseases, and systemic sepsis, and the presence of any type of other malignancy. 

*Laboratory Investigations*

10 milliliters of venous blood were withdrawn from all subjects used for determination of the SNP of *TNF-α *gene *-238G>A rs361525* and *IL-18* gene-*607C>A rs1946518*, complete blood picture, prothrombin time and concentration, random blood glucose, liver function tests including serum total and direct bilirubin, alanine transaminase (ALT), aspartate transaminase (AST), total protein, albumin, alkaline phosphatase (ALP) and Creatinine, urea as for the assessment of kidney function, Serum alpha-fetoprotein (AFP) and HCV Ab and HBsAg were analyzed using automated analyzers according to manufacturer’s instructions. 

*Serum Preparation and DNA Extraction*

DNA extraction was done using GeneJET DNA blood Mini kit-Thermo Fisher Scientific according to kit instructions. 

*Amplification and Real-time PCR allelic discrimination assays*

PCR with sequence-specific primers was used to detect the SNPs of *TNF-α* gene *-238G>A* and *IL-1*8 gene* -607 C>A* in all the studied subjects. Real-time PCR allelic discrimination assays were designed using Taq-Man SNP Genotyping Assays (Applied Biosystems). The SNP Genotyping Assays (40 x) was diluted to a 20x working stock with1×TE buffer, the mixture was vortexed centrifuged, and divided into aliquots in microcentrifuge tubes, each containing 20μl. It was then stored at-20ºC for routine use, as recommended by Applied Biosystems. One Negative Control (NTCs) on each plate was added as recommended by Applied Biosystems, The total volume of each component needed for each assay was calculated, Each well-contained 20μl of reaction mix prepared as follow: 10μl TaqMan Universal PCR Master Mix (2x), 1.0μl of 20x working stock of SNP Genotyping Assay, 4.0μl of DNase-free water, then 5μl of the extracted DNA. The cycling conditions were: AmpliTaq Gold Enzyme Activation10 min at 95°C, Denature15 sec at 92°C, Anneal/Extend for 1 min at 60°C.

*Allelic Discrimination Plate Read and Analysis*

An endpoint plate read was performed After PCR amplification using an Applied Biosystems Real-Time PCR System. The Sequence Detection System (SDS) Software used the fluorescence measurements made during the plate read to plot fluorescence (Rn) values based on the signals from each well. The plotted fluorescence signals indicated which alleles were in each sample an allelic discrimination plate read document was created and set up in the SDS software.

*Statistical analysis*

Quantitative data are summarized as mean **±** SD when normally distributed, and as median (25^th^-75^th^) when abnormally distributed. Differences between groups are detected using analysis of variance (ANOVA) with post hoc test, Kruskal-Wallis, student’s t, and Mann-Whitney as appropriate. Qualitative data are summarized as number (percent) and compared using the Chi-square test. All tests were two-tailed and considered statistically significant at a P-value < 0.05. Statistical analysis is run on SPSS for Mac, release 24 (IBM Corporation, Armonk, NY, USA). 

## Results

A statistically significant association was detected on comparing the mean age of the studied groups with higher mean in the HCC group (P<0.001), the rest of the demographic and clinical data showed no statistically significant association. On comparing different laboratory investigations in the studied groups, AST, ALT, ALP, AFP, urea, and creatinine levels showed statistically significant associations with higher values in the HCC group than HCV group (P= 0.001, 0.001, 0.001, 0.001, 0.008 and 0.042) respectively. However, no statistically significant association was observed when comparing the median values of serum levels of all other parameters.

*Diagnostic Performance of the Polymorphisms*

Three genotypes were recognized for IL18 (CC, CA, AA), homozygous wild, heterozygous and homozygous mutant respectively. Genotypes for TNF-α were (GG, GA, AA), homozygous wild, heterozygous and homozygous mutant respectively. On comparing the frequency distribution of the *SNP -607C>A* in the *IL18* gene among the two studied groups showed statistically significant association (P <0.001) where AA genotype showed higher percentage in HCC group versus HCV group (42.5% vs 12.9%) (P<0.001) as shown in Table 1, However *TNF α* gene polymorphism -238G>A showed no statistically significant association between the two studied groups.

Subgrouping genotypes of *SNP -607C>A* in the *IL18 *gene into two different groups CC/CA versus AA showed that the AA genotype was more frequent in the HCC patient (42.5%) as compared to HCV patient (12.9%). On performing odds ratio on the different genotype subgroups of* IL18* gene *SNP-607C>A*, it was found that individuals harboring the AA genotype were 5 folds riskier to develop HCC compared to those carrying the CC/CA genotypes (OR: 5) (Table 2). However, the sub-grouping of TNF α SNP -238G>A showed no significant association between the two studied groups.

Allele frequency of *IL18 *gene *SNP -607 C>A* showed a significant association between the two studied groups where the A allele was more frequent among the HCC group compared to the HCV group (61.2 % versus 42.9) respectively (P = 0.001). On performing odds ratio, it was found that A allele is a risky allele, where the individuals harboring the A allele were 2.1 folds riskier to develop HCC than those harboring C allele as illustrated in Table 3.

Among all the Laboratory data, only AST, ALT, and ALP showed a statistically significant association between different genotypes of IL18-607 where higher serum levels were associated with the risky genotype AA, (P =0.004, 0.012, 0.001) respectively as shown in Table 4. However, no significant association was found between laboratory data and TNFα-238 genotypes.

Hepatic focal lesions in HCC patients were divided into 3 groups according to their size, < 3cm, 3-5, and >5 cm respectively. There was a statistically significant association between tumor size and different genotypes of *IL18* genes, where 62.5% of patients with tumor size >5 cm carried the risky (AA) genotype. However TNF-α, genotypes showed no statistically significant association with tumor size.

**Table 1:** Frequency Distribution of *TNFα*238 G>A and *IL*18-607 C>A Genotypes in the HCC and HCV Groups

Genotype	HCV (n=70)	HCC (n=80)	P-value
*TNFα*-238G>A			
GG	11 (15.7%)	12 (15%)	0.433
GA	15 (21.4%)	11 (13.8%)	
AA	44 (62.9%)	57 (71.3%)	
*IL18*-607C>A			
CC	19 (27.1%)	16 (20%)	< 0.001
CA	42 (60%)	30 (37.5%)	
AA	9 (12.9%)	34 (42.5%)	

Data are presented as number (percent).

**Table 2:** Subgrouping Genotypes of *SNP* IL18 -607C>A and *TNFα*-238 G>A in HCC and HCV Groups

Genotype:	HCV(n=70)	HCC (n=80)	P-value	OR (95% CI)
*TNFα*238G>A				
GG/GA	26 (37.1%)	23 (28.8%)	0.299	
AA	44(62.9%)	57(71.2%)		
*IL18*-607C>A				
CC/CA	61 (87.1%)	46 (57.5%)	< 0.001	5.0 (2.188-11.47)
AA	9(12.9%)	34(42.5%)		

Data are presented as number (percent).

**Table 3:** Frequency Distribution of Alleles in the HCC and HCV Groups

*TNFα*238G>A	G allele	A allele	P-value	OR (95% CI)
HCC (n=160)	35 (21.9%)	125 (78.1%)	0.357	
HCV (n=140)	37 (26.4%)	103 (73.6%)		
IL18-607	C allele	A allele	P-value	OR (95% CI)
HCC (n=160)	62 (38.8%)	98 (61.2%)	0.001	2.1(1.32-3.34)
HCV (n=140)	80 (57.1%)	60 (42.9%)		

Data are presented as number (percent).

**Table 4:** Association between Clinical and Laboratory Data and Different Genotypes of *TNF-α* 238 G>A and *IL18-*607 C>A

	GG (n= 23)	GA (n=101)	AA (n=26)	P-value
*TNF-α*238 G>A				
AST (IU/l)	76 (50-100)	82 (53-185)	73 (47-120)	0.689
ALT (IU/l)	44 (29-67)	43.5 (28.5-68)	40 (25-70)	0.821
ALP (IU/l)	78 (49-137)	92.5 (56.7-140.5)	95 (70-136.5)	0.392
Urea (mg/dl)	36 (28-67)	40 (30-56)	44 (31-73)	0.837
AFP (ng/ml)	35 (9-250)	21 (6.6-65.3)	34 (8-93)	0.422
	CC (n=35)	CA (n=72)	AA (n=43)	P-value
*IL18-*607C>A				
AST (IU/l)	72 (53-90) ab	66.5 (43.2-97.5) a	93 (61-167) b	0.004
ALT (IU/l)	38 (22-68) ab	38 (23-60) a	56 (32-83) b	0.012
ALP (IU/l)	96 (61-125) a	77 (54.2-98.7) a	130 (88-192) b	<0.001
Urea (mg/dl)	44 (31-64)	50.5 (29.2-79.5)	40 (31-73)	0.978
AFP (ng/ml)	45 (10-86)	21.5 (7.2-71.2)	35 (8-159)	0.338

Data are presented as median (25^th^-75^th^). Groups bearing the same initials are not statistically significant at P = 0.05. AST, aspartate aminotranseferase; ALT, alanine aminotranseferase; ALP, alkaline phosphatase; AFP, alpha-fetoprotein

## Discussion

The first SNP to be discussed is IL-18 -607 C>A, its genotyping revealed that the frequency of the homozygous wild CC, heterozygous CA and homozygous mutant AA genotypes among HCV group were 19(27.1%), 42(60%) and 9(12.9%) respectively versus 16(20%), 30(37.5%) and 34 (42.5%) in the HCC group. On comparing the frequency distribution of the *SNP -607C>A* in the *IL18* gene among the two studied groups it showed significant association (P<0.001) where AA genotype showed higher percentage in HCC group compared to the HCV group (42.5% vs 12.9). Subgrouping was done to genotypes of *SNP -607C>A *in the* IL18* gene into two different groups CC/CA versus AA showed that the AA genotype was more frequent in the HCC group 34(42.5%) versus HCV group 9(12.9%), (P<0.001), On performing odds ratio on the different genotype Subgroups of *IL18* gene S*NP-607C>A*, it was found that individuals harboring the AA genotype were 5 folds riskier to develop HCC compared to those carrying the CC/CA genotypes (OR: 5). Showing that AA genotype could be considered a risky genotype. Allele frequency of *IL18* gene (607 C/A) showed a statistically significant association between the two studied groups where the A allele was more frequent among the HCC group versus HCV group (61.2 % versus 42.9) respectively (P < 0.001). On performing odds ratio, it was found that A allele is a risky allele, where the individuals harboring the A allele were 2.1 folds riskier to develop HCC than those harboring C allele group, (P < 0.001; OR: 2.1). 

In accordance with this study, the study was done by Teixeira et al., (2013) reported a higher frequency of the IL-18 -607⁄A allele with (P = 0.0235) and -607AA genotype with (P = 0.0048 among HCC patients compared to healthy individuals, where the frequency of CA, AA, CC in patient with HCC were 56 (50%), 18 (16%),38 (34%) respectively and that in control group were 105(52%), 12(6%), 85(42%). On the contrary to our study Lau et al., (2016) investigated the association between *SNP -607C>A *in the* IL18* gene and the susceptibility and clinicopathological state of hepatocellular carcinoma. In total, 901 participants, including 559 healthy controls and 342 patients with HCC who were recruited, no statistically significant difference was detected in their study. This difference between their study and ours could be explained by difference in patients’ selection for the study as HBV patients were included in their study while excluded from ours.

The second SNP to be discussed is *SNP -238G>A *in the *TNF-α* gene, its genotyping expressed that the frequency of the homozygous wild GG, heterozygous GA and homozygous mutant AA genotypes among HCV group were 11(15.7%), 15(21.4%), 44(62.9%) respectively versus 12 (15%), 11(13.8%), 57(71.3%) in the HCC group, showing no statistically significant association between the two studied groups. Our results were concomitant with those reported by Shin et al., (2015) their study included 157 HCC patients and 201 control revealing no significant association in the genotype frequencies of the *TNF-α* polymorphism between cases and controls. Also Li et al., (2016) agreed with the result of the current study as they found that the genotype distribution and allele frequencies of *TNF-α-238G>A* polymorphisms did not differ between the HCC group and the control group. Similar to our results, the study done by Yanqiu et al., (2016) who conducted a study in a total number of 175 Chinese subjects were classified into HCC group (102 cases) and healthy control group (73 cases). Serum TNF-α levels and the genotypes of *TNF-α-238G>A SNP* were determined, the genotype and allele frequencies of *TNF-α-238* in HCC group and healthy control groups showed no statistical difference (P>0.05). Results of the present study were in contrast with the meta-analysis done by Cheng et al., (2013) and included eleven case-control studies with a total of 1,572 HCC cases and 1,875 controls. The testing method was the polymerase chain reaction-restriction fragment length polymorphism (PCR-RFLP), which was used in 8 of those 11 studies they reported that the *TNF α -238 G>A* polymorphism was significantly associated with increased risk of hepatocellular carcinoma. This contrast may be due to the difference of the gene distribution in different geographical populations or due to difference in methodology of detecting the SNP, however it was opposed by another meta-analysis done by Hu et al., (2014) included ten studies concluded that there is no association found between *TNF α-238G>A *polymorphism and risk of HCC. On the contrary to our results the study done by Teixeira et al., (2013) which was conducted on a total number of 314 Brazilian subjects they were divided into two groups, Group (1) Included 112 patients with hepatocellular carcinoma, group (2) included 202 healthy controls, they reported that TNFα -238/A allele showed significant susceptibility to HCC with (P = 0.0235), and that genotypes *TNFα -238G>A* were more frequent among patients with HCC compared to controls with (P = 0.0011). This discrepancy may be due to a racial difference between the populations also environmental and genetic factors should be considered because HCC is a multifactorial disease.

In the current study, hepatic focal lesions in HCC patients were divided into 3 groups according to their size, < 3 cm, 3-5, and >5 cm respectively. There was a statistically significant association between tumor size and different genotypes of *IL18* genes, where 62.5% of patients with tumor size >5 cm carried the risky (AA) genotype, however TNF α genotypes showed no statistically significant association with tumor size. In a similar study Teixeira et al., (2013) reported that neither TNFα nor IL-18 genotypes showed statistically significant association with tumor size although TNFα -238/A allele was slightly more frequent in tumors larger than 10 cm compared to tumors smaller than 5 cm (P = 0.0889).

In Conclusion, detection of *SNP -607C>A* in* IL18 *gene showed statistically significant association with the development of HCC, where AA genotype and A allele showed higher frequency in HCC group, suggesting its potential use as a diagnostic non-invasive tool complementary to AFP that may allow identifying a new group of HCC patients at an earlier stage. However, we didn’t find relevant results regarding the SNP of TNF-α 238G>A associated with HCC.

## Author Contribution Statement

None.
